# The effect of laser therapy for the treatment of dentin hypersensitivity on surface roughness and bacterial adhesion

**DOI:** 10.1007/s10103-024-04166-0

**Published:** 2024-08-09

**Authors:** Ozge Parlar Oz, İrem Karagozoglu, Ipek Kocer, Nermin Demırkol, Yasemin Zer

**Affiliations:** 1 Faculty of Dentistry, Department of Prosthodontics, Gaziantep University, Gaziantep, Turkey; 2 Faculty of Medicine, Department of medical microbiology, Gaziantep University, Gaziantep, Turkey; 3 Faculty of medicine, Department of medical microbiology, Sanko University, Gaziantep, Turkey

**Keywords:** Dentin hypersensitivity, Bacterial adhesion, Diode laser, Nd:YAG laser, Er,Cr:YSGG laser

## Abstract

The aim of the study was to measure the degree of dentine surface roughness caused by five distinct lasers used to treat dentine hypersensitivity, as well as to evaluate the subsequent bacterial colonization on these irradiated surfaces. Sixty human maxillary premolar teeth without caries or restoration which were extracted for periodontal reasons were used in this study. Five different types of lasers were applied to the root dentin surface. Tested samples were divided into six groups of 10 samples each; control, diode (810 nm), diode (980 nm), Nd: YAG, Er: YAG, and Er, Cr: YSGG laser groups. The arithmetic mean of the surface roughness values (Ra) and the average roughness over a measurement area (Sa) were measured pre- and post-application using any of the laser types. Swab samples were then collected from the dentin surface. Following a 24-hour incubation period at 37 °C, the colony forming units were counted using a stereoscope. The results demonstrated a statistically significant difference in the surface roughness values pre- and post-application (Ra and Sa, respectively) in the Er, Cr: YSGG laser group (*p* = 0.037,*p* = 0.007). No significant difference was observed in the other groups (*p* > 0.05). There was no statistically significant difference in the number of bacterial colonies observed between the test and control groups. Diode and Nd: YAG lasers showed either a decrease or no change in surface roughness; however, the hard tissue lasers (Er: YAG, Er, Cr: YSGG) showed an increase. The Er: YAG and Nd: YAG laser groups exhibited decreased bacterial adhesion compared to the other groups.

## Introduction

Due to the increased awareness of oral and dental health in our world today, as well as the development of preventative and more accessible treatment methods, the longevity of teeth in the oral cavity has improved. Teeth that remain in the mouth for a longer period of time are subjected to increased abrasion, resulting in the exposure of the dentin surface. In addition, the dentin tubules become exposed to the oral environment as a result of parafunctional movements like clenching and grinding, improper tooth brushing habits, excessive consumption of acidic foods, and the use of mouthwashes. Dentin hypersensitivity, resulting from the exposure of dentinal tubules, can create physical and psychological issues in affected individuals and significantly reduces their quality of life. Under normal conditions, the dentin surface is protected by either the enamel or cementum and is not sensitive to direct stimulation; dentin hypersensitivity occurs when the dentin surface becomes exposed due to abrasion of the enamel or cementum [1]. Dentin hypersensitivity is characterized as an acute, sudden, sharp, and transient toothache that occurs on the dentin surface, which is caused by any stimuli and cannot be attributed to any underlying pathology [2]. Pain often arises from mechanical, thermal, or chemical stimuli on dentin-exposed surfaces, and ceases once the stimulus is no longer present.

Several theories have been proposed to explain the occurrence of dentin hypersensitivity. The prevailing consensus is that stimulus leads to the displacement of fluid within the dentinal tubules. The hydrodynamic theory explains that pain is caused by the movement of fluid within and outside the tubules, which stimulates the nerve endings at the pulp-dentin interface [3]. Therefore, the primary treatment for dentin hypersensitivity is to reduce fluid movement and dentin permeability. The closure of exposed dentin tubules can potentially decrease the occurrence of dentin hypersensitivity; however, several studies have indicated that dentin hypersensitivity may still persist despite successful closure of the tubules [4–6].

Various methods and materials have been developed to address dentin hypersensitivity. Traditional methods for addressing tooth sensitivity include use oftoothpastes, professional desensitizers, fluoride applications, varnishes. Typically, various agents that decrease or modify nerve impulses are used, either by expert application or local administration at home. Potassium salts, such as potassium fluoride, potassium chloride, and the most commonly used potassium nitrate, stand out as nerve modifiers [7]. Sodium fluoride gel (NaF) is the most commonly employed tubular occlusion agent [8]. The process relies on the mechanical occlusion caused by the precipitation of calcium fluoride crystals that are insoluble and do not adhere to the tubules. Therefore, it is unable to withstand the pressures of the oral environment and its efficacy diminishes over time [9]. Certain treatment options such as the addition of desensitizers to toothpastes, aim to occlude the dentinal tubules in order to reduce sensitivity.

Other alternatives in reducing dentin hypersensitivity include laser therapy. Studies have demonstrated the clinical efficacy from using various lasers for therapy [9–11]. Er: YAG and Er, Cr: YSGG lasers can occlude dentinal tubules by generating elevated temperatures in the tissue [12, 13]. Nd: YAG, GaAlAs and He-Ne lasers interfere with the Na+-K + ion pump in the cell membrane and prevent the transmission of the pain signals, thereby reducing pain symptoms [13, 14].

Laser therapy for dentin hypersensitivity is a convenient and quick method for patients. The impact is immediately noticeable following a single application. The effect can last for an average of 3–6 months with no reported side effects [15]. While laser desensitisation is an effective method, it does come with several drawbacks. It has been reported in the literature that lasers result in the roughening of the tooth surface [16, 17]. Bacteria colonise the tooth surface and form a biofilm through continuous adhesion. The initial adhesion of bacteria to the tooth surface is a crucial step in the development of bacterial plaque. The irregular geometry of a rough surface provides a better environment for bacteria to engage with the surface and establish a strong attachment [18, 19]. In contrast, a rough surface can result in the accumulation of bacteria, leading to the development oftooth decay and gum disease [20, 21]. The aim of this study was to assess the surface roughness and bacterial adhesion on dentin caused by different lasers used in the treatment of dentin hypersensitivity. The null hypothesis posited that there would be no changes in surface roughness and bacterial retention following dentine hypersensitivity treatments.

## Materials and methods

### Sample preparation

**Fig. 1:**
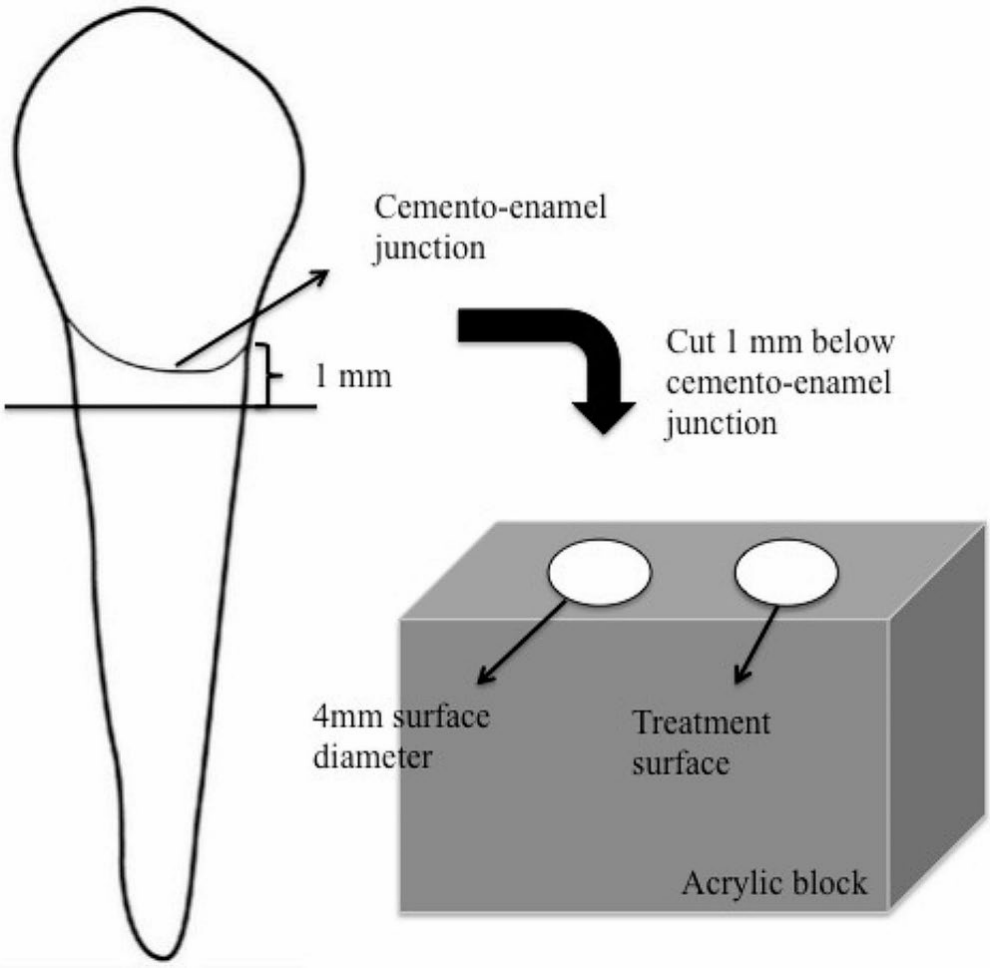
Diagram of the tooth set-up

Prior to the commencement of the study, ethical approval was obtained from the Gaziantep University Clinical Research Ethics Committee (decision number: 2019/508). The minimum number of teeth required to achieve a statistically significant strong correlation (*r* = 0.80) between the amount of surface roughness and bacterial growth was found to be 9 (α = 0.05, 1-β = 0.80). Power analysis was performed using G-Power version 3.1.9.2. Sixty human maxillary premolar teeth without caries or restoration which were extracted for periodontal reasons were used in the study. The teeth were sectioned 1 mm below the cemento-enamel junction using a diamond saw (Isomet Diamond Wafering Blade, Buehler, Lake Bluff, IL, USA) in a low-speed, water-cooled cutting device. The root portion of the teeth were embedded in acrylic blocks to facilitate the execution of the treatments. Figure 1 shows that a total of 30 acrylic blocks were obtained by placing 2 teeth in each block. The fine abrasive paper (600, 800 and 1200 nm; 3 M ESPE, St. Paul, Minn) was used to smoothen one side of each sample by a single operator for 60 s. A standardised region with a diameter of 4 mm was established to facilitate the application of surface treatments and enable easier detection of surface roughness and bacterial adhesion.

### Surface roughness test

The created blocks were immersed in distilled water. Each tooth was subsequently treated with a 24% ethylenediaminetetraacid (EDTA) solution for 60 s. Each sample was then purified using distilled water. This procedure opened the dentinal tubules, imitating the appearance of exposed dentin [20]. The surface roughness values were measured prior to any dental treatment. This measurement was done using an optical, non-contact, 3D surface profilometer (ZeeScope Optical Profilometer). Ra and Sa values were recorded. Following laser treatments, the surface roughness was reassessed using the same protocol.

### Group formation and laser procedures

The blocks and teeth were randomly divided into six groups. The randomization process was conducted using the freely available ‘Research Randomizer’ website specifically developed for researchers. These groups are: control, diode (810 nm), diode (980 nm), Nd: YAG, Er: YAG, and Er, Cr: YSGG laser groups. The groups and processes for laser application are listed below and shown in Table 1.

- Group 1: Control group, no surface treatment following tooth preparation.
- Group 2: Diode laser (810 nm wavelength) (Cheese dental laser; Wuhan Gigaa Optronics Technology Co. Ltd., Wuhan, China) was applied to surface using a 300 micron fibre optic cable with spot mode and a power of 0.2 W in continuous mode for 30 s. [22].
- Group 3: Diode laser (980 nm wavelength) (Cheese dental laser; Wuhan Gigaa Optronics Technology Co. Ltd., Wuhan, China). A power of 0.5 W was applied in continuous mode for 60 s, T on at 100 ms and T off at 100 ms, using a 300 micron diameter fibre tip [23].
- Group 4: Nd: YAG laser (1064 nm wavelength) (Fotona Laser AT, Fidelis Plus III, Ljubljana, Slovenia)was applied according to the parameters recommended for Fotona laser devices and based on previous investigations in the literature [23].The power indicator was set to 1 W, the frequency was set to 10 Hz, and the device was set to short pulse mode (pulse duration, 180 µs). A 300 μm Nd: YAG fibre was used for 60 s (the distance between tissue and fibre tip was 2 mm), applied slowly and evenly to the 4 mm surface [24].
- Group 5: Er: YAG laser (2940 nm wavelength) (Fotona Laser AT, Fidelis Plus III, Ljubljana, Slovenia) was applied according to the parameters recommended for Fotona laser devices and studies in the literature [23]. An R02-handpiece (Fotona AT, Fidelis III, Ljubljana, Slovenia) was positioned 6 mm away from the tissue and used for laser application for 60 s with power display 0.2 W, energy per pulse 80 mJ/pulse, frequency 3 Hz and the device set to short pulse mode (pulse duration 300 µs). The treatment was administered gradually and evenly with water irrigation (25 mL/min, to protect from thermal changes) to the surface (surface diameter, 4 mm) [24].
- Group 6: Er, Cr: YSGG laser (2780 nm wavelength) (Waterlase, Biolase Technology, Irvine, CA). The application procedure: An Er, Cr: YSGG (A Z6 sapphire tip, 6 mm length, 600 μm diameter) laser is applied perpendicularly to the surface with a 1-mm defocused beam over an area of 4 mm for 60 s with scanning movements, 0% water, and 0% air [25]. The samples were subjected to the following parameters; frequency 20 Hz, pulse width 140–200 µs, and power 0.25 W [25].

Following the laser operations, the samples were rinsed with distilled water for 10 s and were subsequently preserved in sterile distilled water.

**Table 1:** Lasers sytems and parameters

Lasers	Parameters	Manufacturers
Diode (810 nm)	300 micron diameter fibre, a power of 0.2 W, continuous mode, 30 s.	(Cheese dental laser; Wuhan Gigaa Optronics Technology Co. Ltd., Wuhan, China)
Diode (980 nm)	300 micron diameter fibre, a power of 0.5 W, continuous mode, 60 s, T on at 100 ms and T off at 100 ms,	(Cheese dental laser; Wuhan Gigaa Optronics Technology Co. Ltd., Wuhan, China)
Er, Cr: YSGG	A Z6 sapphire tip, 6 mm length, 600 μm diameter, frequency 20 Hz, pulse width 140–200 µs, a power of 0.25 W, 60 s.	(Waterlase, Biolase Technology, Irvine, CA)
Er: YAG	A R02-handpiece, non-contact mode, a power of 0.2 W, energy per pulse 80 mJ/pulse, frequency 3 Hz, short pulse mode, 60 s	(Fotona Laser AT, Fidelis Plus III, Ljubljana, Slovenia)
Nd: YAG	A 300 μm Nd: YAG fibre, a power of 1 W, the frequency 10 Hz, short pulse mode, 60 s.	(Fotona Laser AT, Fidelis Plus III, Ljubljana, Slovenia)

### Bacterial adhesion test

First, the samples were sterilised. The bacterial adhesion test utilized *Streptococcus mutans* that was cultivated in sucrose medium. Dentin samples were placed in sterile cell culture plates and inoculated overnight with standard cultures in the same medium supplemented with 5% sucrose. The samples were then incubated at 37 °C for 24 h and covered with 1.5 mL of Brain Heart infusion.

Following the incubation period, the dentin fragments were washed in sterile distilled water to remove non-adherent microorganisms. Swab samples were then collected from the dentin surface and evenly distributed onto the surface of agar plates supplemented with sucrose and sheep’s blood. Following a 24-hour incubation period at 37 °C, colony forming units (CFU) were counted using a stereoscope and results were expressed in CFU/mL.

### Statistical analysis

Kolmogorov-Smirnov and Shapiro-Wilk tests were used to test if the distribution of the data deviated from the normal distribution. Kruskal-Wallis and Dunn multiple comparison tests were used to compare non-normally distributed data across the six groups. A Wilcoxon test was performed to compare initial and final measurements. The statistical analysis was performed using SPSS for Windows version 24.0 and *p* < 0.05 was considered statistically significant.

## Results

The bacterial colonization and surface roughness values of the tested groups were subjected to statistical analysis. The surface roughness values were summarized in Table 2 using descriptive statistics.

**Table 2:** Descriptive statistics for surface roughness values

	Surface Roughness (Ra)			Surface Roughness (Sa)		
	Initial	Final	P	Initial	Final	P
Control	0.04 ± 0.01	0.04 ± 0.01	1.000	0.06 ± 0.01	0.06 ± 0.01	1.000
Diode(810 nm)	0.05 ± 0.03	0.04 ± 0.02	0.445	0.09 ± 0.03	0.08 ± 0.02	0.445
Diode(980 nm)	0.06 ± 0.04	0.04 ± 0.01	0.139	0.1 ± 0.05	0.07 ± 0.02	0.169
Er, Cr: YSGG	0.05 ± 0.02	0.08 ± 0.04	0.037*	0.07 ± 0.02	0.1 ± 0.05	0.007*
Er: YAG	0.04 ± 0.01	0.04 ± 0.02	0.241	0.07 ± 0.02	0.07 ± 0.02	0.799
Nd: YAG	0.05 ± 0.03	0.05 ± 0.02	0.646	0.08 ± 0.04	0.07 ± 0.03	0.203
P*	0.397	0.010*		0.213	0.021*	

*Significant at 0.05 level; Wilcoxon test for within group, Kruskal Wallis test for between group comparisons

The results showed a statistically significant difference in the surface roughness values (Ra and Sa) between the pre and post-application stages in the Er, Cr: YSGG laser group (*p* = 0.037,*p* = 0.007). No statistically significant difference was seen in the other groups (*p* > 0.05).

**Fig. 2:**
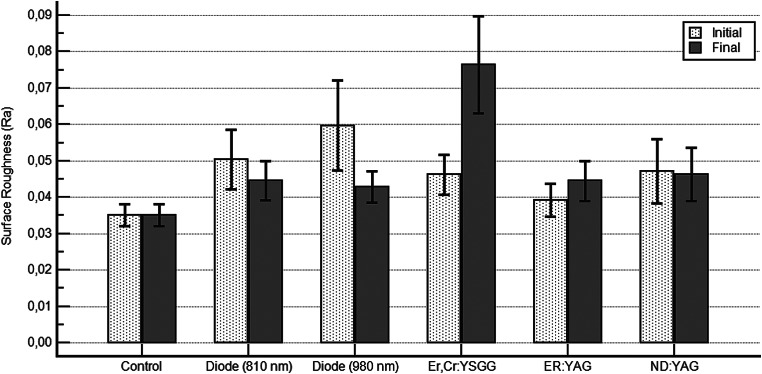
Comparison of final RA values between groups (Dunn’s test)

The Ra values resulting from the laser applications were compared and summarised in Fig. 2. Statistically significant differences were observed between the control and Er, Cr: YSGG laser groups (*p* = 0,001); the Nd: YAG and Er, Cr: YSGG laser groups (*p* = 0,009); the diode (980 nm) and Er, Cr: YSGG laser groups (*p* = 0,011); the Er: YAG and Er, Cr: YSGG laser groups (*p* = 0,015); and the diode (810 nm) and Er, Cr: YSGG laser groups (*p* = 0,015).

**Fig. 3:**
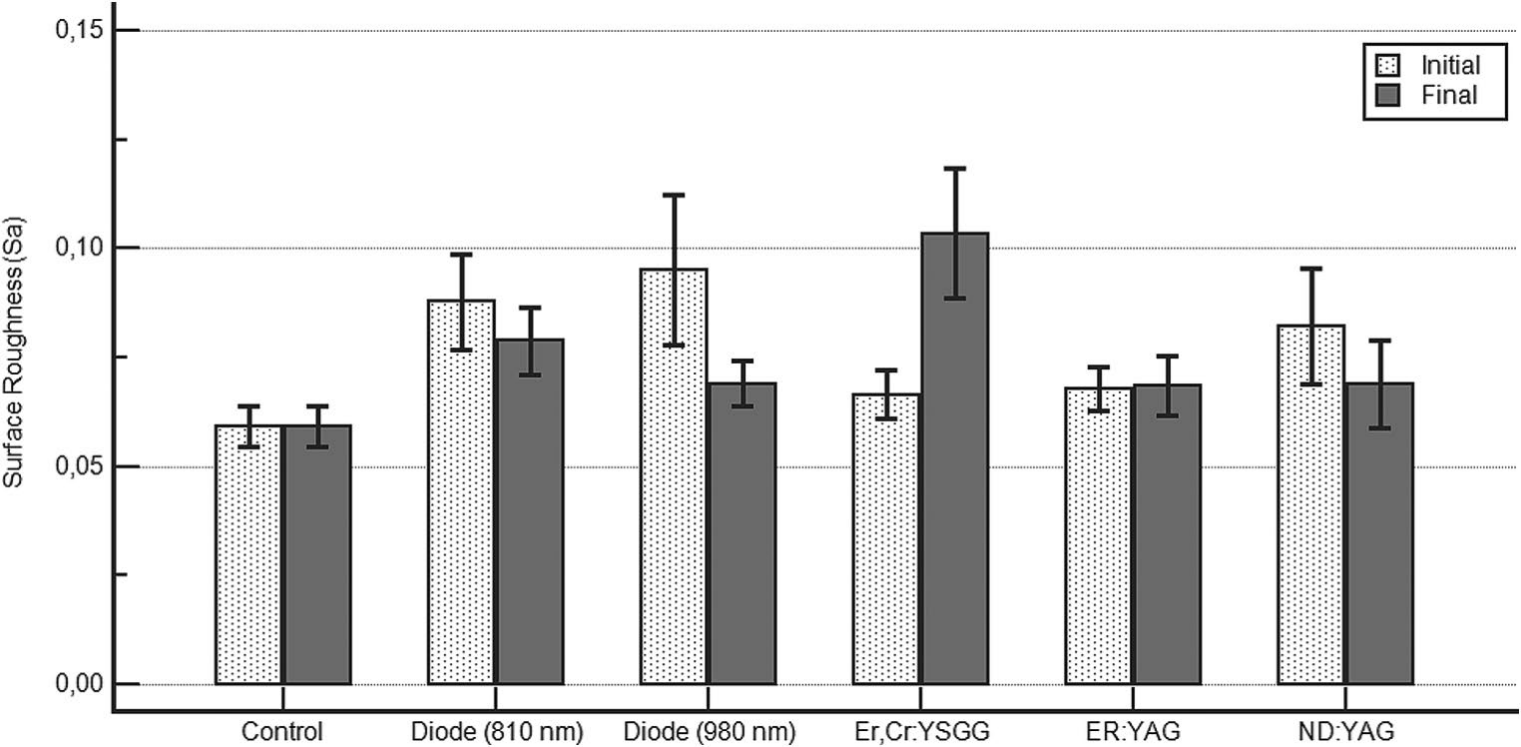
Comparison of final SA values between groups (Dunn’s test)

The Sa values resulting from the laser applications were compared and summarised in Fig. 3. Statistically significant differences were observed between the control and Er, Cr: YSGG laser groups (*p* = 0,001); the control and diode (810 nm) laser groups (*p* = 0,034); the Nd: YAG and Er, Cr: YSGG laser groups (*p* = 0,007); the diode (980 nm) and Er, Cr: YSGG laser groups (*p* = 0,037); and the Er: YAG and Er, Cr: YSGG laser groups (*p* = 0,042).

The average CFU/mL obtained in all the test groups are shown in Table 3. There was no statistically significant difference in the number of bacterial colonies seen between the test groups and the control group. The Er: YAG and Nd: YAG laser groups presented reduced bacterial adhesion compared to the other groups.

**Table 3:** The average CFU/mL obtained in all test groups

	Colony amount (cfu/ml)	P
Control	17,000 ± 10593,5	0.133
Diode(810 nm)	8000 ± 14757,3	
Diode(980 nm)	8000 ± 13165,61	
Er, Cr: YSGG	9000 ± 12866,84	
Er: YAG	7000 ± 9486,83	
Nd: YAG	7000 ± 6749,49	

## Discussion

Laser systems used to treat dentin hypersensitivity are an effective treatment option, but they have a tendency to induce tooth surface roughness, which can lead to bacterial retention. In this study, 5 different types of lasers with varying wavelengths were compared and only the Er, Cr: YSGG laser resulted in an increase in roughness on the root dentin surface. Since the wavelength of the Er, Cr: YSGG laser (2780 nm) is close to the wavelength of the Er: YAG laser (2940 nm), there was an increase in surface roughness in the Er: YAG laser group. However, this increase was not deemed statistically significant.

Laser treatments are the preferred method over chemical agents for treating dentin hypersensitivity due to their ease of application and longer duration of action [17]. It has been reported that chemical agents used in conventional dentin hypersensitivity treatments have been found to cause surface roughness [20]. Nogueira et al. reported that roughness and bacterial adhesion were significantly higher on flouride varnish-treated surfaces compared to laser-treated surfaces [21]. In a study, it was reported that all chemical agents and laser treatments that were examined were found to decrease dentin permeability [17]. Upon evaluating studies investigating the impact of conventional treatments and lasers on surface roughness, it can be concluded that chemical agent applications cause more roughness. The results of this study indicate that only the Er, Cr: YSGG laser increased the surface roughness compared to the pre-application condition.

When considering treatments for dentin hypersensitivity, it is important to evaluate both the benefits and the potential risks. An inherent danger of dentin hypersensitivity treatments is the possibility of causing dentin surface roughness and associated caries formation. Consequently, in vitro studies are gaining significance in this context. Several in vitro studies have have investigated the impact of chemical agents and laser systems on tooth surface roughness. However, there is no precise regarding the level of surface roughness induced by different laser systems and the types of bacteria and biofilm that adhere to teeth [16, 20, 21]. In this study, only the Er, Cr: YSGG laser exhibited a significantly increase in surface roughness and bacterial colonisation in comparison to the control and other tested groups. This finding was related to the fact that hard tissue lasers can cause morphological and chemical changes in dental hard tissue. There is a lack of comparative research in the existing literature that examine all types of lasers. A comparative investigation on high intensity lasers reported that Er, Cr: YSGG laser application resulted in more surface roughness compared to diode and Nd: YAG lasers, which aligns with our own findings [21]. A recent study examined the efficacy of Er: YAG laser by applying at different intensities [16]. The study found that as the energy density increased, the roughness of the enamel surface also increased [16]. Additionally, the study reported that higher surface roughness leads to greater bacterial adhesion [16]. Similarly, in our study, bacterial colonisation was highest in the Er, Cr: YSGG laser group in which the surface roughness increased the most.

Clinical success with laser treatment of dentin hypersensitivity has been reported in many in vivo studies [15, 22, 25]. Low-intensity lasers are known to be effective in reducing dentin hypersensitivity, but the effect of high-intensity lasers is still a subject of debate. The Er: YAG laser’s high wavelength absorption in water can cause the evaporation of the dentinal fluid and smear layer [26, 27]. Studies evaluating the efficacy of Er: YAG and Er, Cr: YSGG lasers have reported that these lasers are highly effective in treating dentinal sensitivity. They exhibit the best clinical performance, do not damage the pulp, and remain effective for 6 months [27, 28]. High-intensity lasers are used to treat dentin tubules by coagulation, protein precipitation, or the formation of insoluble calcium complexes, which reduce or close the diameter of the tubules. Clinically, the outcome may be deemed satisfactory. However, based on the findings of our laboratory investigation, it can be concluded that high-intensity lasers result in greater surface roughness, whereas low-intensity lasers are more reliable in clinical use.

Currently, dentin hypersensitivity treatment involves the utilization of different types of lasers, different wavelengths, chemical agents, or a combination of these approaches. A recent systematic review has determined that both the Nd: YAG and the diode lasers are effective in the treatment of dentin hypersensitivity [29]. Furthermore, according to our study findings, Nd: YAG and diode lasers had the least effect on surface roughness. Therefore, it can be said that these two types of laser are more preferable in clinical conditions and are the least harmful options for the tooth.

Laser effects are influenced by several parameters such as wavelength, output power, duration and mode of emission, beam profile, and spot size [30]. Typically, diode lasers utilize a wavelength range of 810–980 nm. While 808–810 nm wavelength was used in some studies [31–35], 980 nm wavelength was used in other studies [23, 36–38]. However, we do not have clear evidence regarding the optimal wavelength for diode lasers or the best approach to achieve photobiomodulatory effects or superficial melting and dentinal tubule occlusion. Therefore, diode lasers with both wavelengths were used in our study. There was no difference in the surface roughness and bacterial colonisation between the two diode lasers.

In surface roughness analysis, the Ra value, typically representing the arithmetic mean, has been considered. Cury et al. [20] measured the Sa value of surface roughness. The Ra value (roughness average) represents the mean of the surface heights along a measurement path. Sa value is the average roughness within a specified measurement area. Therefore, in order to obtain more accurate data, we measured both Ra and Sa values in our study. Similar data were obtained for both values.

The present study was carried out on extracted human teeth inside a controlled laboratory environment. The aim of the study is to measure surface roughness, and it is not possible to measure surface roughness in the oral environment. However, bacterial colonisation in the oral environment could differ under in vitro conditions, and this may be considered a limitation of the study.

## Conclusions

The present study was conducted using five different laser types and wavelengths. It was concluded that only the Er, Cr: YSGG laser resulted in an increased surface roughness and bacterial colonisation. There was also an increase in surface roughness for the Er: YAG laser group, but it was not statistically significant. The Er: YAG and Nd: YAG laser groups exhibited lower bacterial adhesion compared to the other groups. Diode lasers and Nd: YAG laser showed a decrease or absence of alteration in surface roughness, while hard tissue lasers (Er: YAG, Er, Cr: YSGG) demonstrated an increase. Results of this in-vitro study will be useful for clinicians to evaluate the selection of the appropriate laser type for the treatment of dentin hypersensitivity.
